# The clinical utility of tuberculin skin test and interferon-γ release assay in the diagnosis of active tuberculosis among young adults: a prospective observational study

**DOI:** 10.1186/1471-2334-11-96

**Published:** 2011-04-18

**Authors:** Ji Eun Lee, Hee-Jin Kim, Sei Won Lee

**Affiliations:** 1 Department of Internal Medicine, Armed Forces Capital Hospital, Seongnam-si, Gyeonggi-do, Republic of Korea; 2 The Korean Institute of Tuberculosis, Seoul, Republic of Korea; 3 Department of Internal Medicine, Seoul National University Bundang Hospital, Seongnam, Republic of Korea

## Abstract

**Background:**

The roles of the tuberculin skin test (TST) and QuantiFERON^®^-TB Gold In-Tube assay (QFT-IT) in the diagnosis of active tuberculosis (TB) are not clear in young adults. We evaluated the diagnostic accuracy of the TST and QFT-IT in smear-negative TB among young adults with no underlying disease.

**Methods:**

We prospectively enrolled 166 young participants 20-29 years of age with suspected active TB in a military hospital of South Korea. The TST and QFT-IT were performed for all participants.

**Results:**

Of the 143 patients included in the analysis, active TB was diagnosed in 100 (69.9%). There were 141 male patients, none of whom had immunosuppressive disease. The sensitivity, specificity, positive predictive value (PPV), and negative predictive value (NPV) of TST were 94% (95% CI, 87-98%), 88% (95% CI, 74-96%), 95% (95% CI, 88-98%), and 86% (95% CI, 72-94%), respectively. The sensitivity, specificity, PPV, and NPV of the QFT-IT were 93% (95% CI, 86-97%), 95% (95% CI, 81-99%), 98% (95% CI, 92-99%), and 84% (95% CI, 69-93%), respectively. No significant differences were found between the TST and QFT-IT in any statistic.

**Conclusions:**

Both the TST and QFT-IT showed high sensitivity and specificity in differentiating active TB from other diseases. The diagnostic accuracy of these two tests did not differ significantly when applied to this clinical population of young, immunocompetent adults in whom neonatal BCG vaccination was common, there was no history of previous TB and in whom suspicion of TB was high.

**Trial registration:**

ClinicalTrials.gov: NCT00982969

## Background

Tuberculosis (TB) is an important public health problem worldwide. In 2007, there were 9.27 million cases of TB, and 1.3 million deaths occurred among HIV-negative TB cases [1]. South Korea has an intermediate TB burden; the incidence of new cases is 90 per 100 000 people per year despite an intensive effort for disease control [2]. A prompt and accurate diagnosis is critical for effective control and management of TB, but conventional diagnostic methods have their limitations. Completion of mycobacteria culture takes 3-8 weeks on solid medium and 7-21 days in liquid medium [3,4]. The sensitivity of acid-fast bacilli (AFB) smears is low [3]. The polymerase chain reaction test is often used for rapid microbiological diagnosis of TB, but its sensitivity for smear-negative respiratory samples ranges from 48 to 53% [3,5,6].

The tuberculin skin test (TST) and whole-blood interferon-γ release assay (IGRA) have been employed for diagnosing latent TB infection (LTBI), but they have also been investigated for their usefulness in the diagnosis of active TB. Although the IGRA is considered to be more useful than the TST in differentiating active TB [7-9], it has not been shown to be adequate for use as a rule-out test for active TB, with a pooled sensitivity and specificity of 80% [10]. However, in some populations, a higher sensitivity and specificity of more than 90% were reported [11,12], which suggests that the IGRA may be utilized in selected populations. The TST has low specificity in Bacille Calmette-Guérin (BCG)-vaccinated populations [13,14] and several disadvantages compared with IGRA, but it is inexpensive and simple, facilitating its use in the community and in resource-limited settings [15].

Young adults have some distinctive characteristics. They are immunologically active and have a shorter duration of unknown TB exposure than do elderly people. Furthermore, nontuberculous mycobacteria (NTM) sensitization is not prevalent in this group, and more than 10 years have passed since their BCG vaccination at birth. The clinical utility of the TST and IGRA may differ by age, and the rates of positive TST results were found to be significantly higher in younger than in elderly patients (70% vs. 27%, respectively) [8]. We evaluated the diagnostic accuracy of the TST and IGRA in the diagnosis of smear-negative TB in young and immunocompetent adults in the Korean military, where the IGRA is not usually available.

## Methods

### Participants and data collection

We prospectively enrolled all individuals 20 to 29 years old who were highly suspect for TB at the Armed Forces Capital Hospital, a central military referral hospital in Korea, between May 2008 and September 2009. Subjects who had a previous history of TB or who had taken anti-TB medication for more than 1 month before the visit were excluded. After giving informed consent, each participant was asked to complete a questionnaire about his or her demographics, previous history of TB, smoking status, and other factors. In addition, the TST and QuantiFERON^®^-TB Gold In-tube assay (QFT-IT; Celletis Ltd., Victoria, Australia) were performed on all participants. This study was reviewed and approved by the Institutional Review Board of the Armed Forces Medical Command.

### Tuberculin skin test

The TST was performed after blood sampling for QFT-IT by trained personnel following standard procedures. For this test, 0.1 mL (2 TU) purified protein derivate (RT23; Statens Serum Institute, Copenhagen, Denmark) was injected intradermally into the inner side of the left forearm by three experienced nurses. Transverse induration at the TST site was measured in mm after 48-72 h by an experienced physician (Dr. S. W. Lee). We defined a positive test as an induration of ≥10 mm [16].

### Interferon-gamma release assay

QFT-IT, a type of IGRA, was performed according to the manufacturer's instructions. Blood samples were collected in three special tubes: one coated with the *Mycobacterium tuberculosis *(MTB)-specific peptides ESAT-6, CFP-10, and TB 7.7 (Rv2654, only peptide 4); one with mitogen as a positive control; and one without antigen as a negative control. Within 8 h of blood sampling, tubes were incubated for 24 h at 37°C, followed by centrifugation and cold storage until testing, as specified by the manufacturer. The concentration of IFN-γ in plasma was measured using a commercial QFT-IT ELISA. The test result was determined as negative, intermediate, or positive (cutoff at 0.35 IU/mL and ≥25% of the nil control) by the manufacturer's software. The doctors in charge were blinded to the QFT-IT result prior to diagnosis.

### Definitions and diagnoses

Final diagnoses were made on the basis of all clinical, radiologic, microbiologic, and pathologic information.

Active TB cases were defined as follows:

#### Active pulmonary TB

Active pulmonary TB was defined if: (i) MTB was cultured from sputum, bronchial specimens or (ii) patient data met the definition of a clinical case of TB, according to the WHO [17]. For clinical cases of TB, radiographic lesions compatible with active TB upon HRCT were mandatory. Active pulmonary TB was defined as the presence of cavities, branching linear opacity, or multiple non-calcified nodules upon HRCT. However, lesions that appeared mainly as calcified nodules or fibrotic bands were excluded from the diagnosis of active TB [17-19]. In participants with lesions that suggested active TB, but who did not have positive acid-fast smears or culture of *M. tuberculosis*, broad-spectrum antibiotics (combination of a third-generation cephalosporin and a macrolide) were prescribed for 1 week. If the lesions on chest X-ray (CXR) or HRCT showed definite improvement, a diagnosis of active TB was excluded.

#### Pleural TB

Pleural TB was diagnosed if: (i) *M. tuberculosis *was detected in the culture of pleural fluid or tissue, or (ii) exudative pleural effusion showed lymphocyte predominance, negative cytology, low carcinoembryonic antigen (< 5 ng/ml) and high adenosine deaminase (≥50 IU/l).

#### Lymph node TB

Lymph node TB was diagnosed if one of the following criteria: (i) detection of *M. tuberculosis *in a culture or of lymph node tissue, (ii) pathological changes consistent with TB or, (iii) positive real-time polymerase chain reaction for *M. tuberculosis *in the tissue of lymph nodes confirmed by commercialized kit (COBAS^® ^TaqMan^® ^MTB test, Roche, West Sussex, UK) and a distinct clinical response to a anti-TB medication.

The diagnosis of other types of extrapulmonary TB was made prospectively on the basis of clinical manifestation consistent with TB and cultures of *M. tuberculosis*.

Cases were defined as non-TB only when another definite diagnosis had been made.

#### Pneumonia

Pneumonia was defined as a new pulmonary infiltrate associated with at least one of the following factors: a new or increased cough, abnormal temperature (<35.8°C or >37.8°C), or an abnormal leukocyte count (leukocytosis, leukopenia, or the presence of immature neutrophils). We confirmed the resolution of the pulmonary infiltrate after administrating broad-spectrum antibiotics.

#### Pulmonary paragonimiasis

Pulmonary paragonimiasis was diagnosed by detection of parasite ova in sputum and/or stools. In egg-negative cases with peripheral eosinophilia and suspicious radiologic findings including patchy densities, cavities, pleural effusions, and ring shadows, elevated serum specific IgG antibody concentration for *Paragonimus westermani *helped to confirm the diagnosis.

#### Empyema

Empyema refers to a grossly purulent pleural effusion associated with pneumonia.

### Statistical methods

Using active TB as the gold standard, we classified the results of TST and QFT-IT as true positive (TP), false negative (FN), false positive (FP), or true negative (TN). The sensitivity (TP/[TP + FN]), specificity (TN/[TN + FP]), positive predictive value (PPV) (TP/[TP + FP]), negative predictive value (NPV) (TN/[TN + FN]), positive likelihood ratio (LR+) (sensitivity/[1 - specificity]), and negative likelihood ratio (LR-) ([1 - sensitivity]/specificity) were calculated for each diagnostic test. The area under the curve (AUC) of the receiver operating characteristic curve (ROC) was calculated to measure the overall accuracy of the test. Ninety-five percent confidence intervals (CIs) were estimated according to the binominal distribution. A χ^2 ^test was used to compare the positive *p*-values, for which <0.05 was considered significant. Concordance between test results from the TST and QFT-IT was assessed using κ coefficients [20]. Statistical analyses were performed using STATA 10.1 software (STATA Corp., College Station, TX, USA).

## Results

### Baseline characteristics of the study populations

A total of 166 patients were recruited, 23 of whom were excluded from the final analysis because their sputum smear results were positive. All patients were HIV negative, and no patient had risk factors for immunosuppression. A BCG scar was present in 102 patients (71.3%). Active TB was diagnosed in 100 (69.9%) patients. Forty-three patients (30.1%) were classified as non-TB cases. Pneumonia was the most common diagnosis among non-TB cases. Compared to non-TB cases, TB cases had a significantly higher percentage of patients with sputum. Concerning the laboratory data, TB cases had a significantly lower peak body temperature and serum CRP concentration (Table 1).

**Table 1:** Baseline characteristics of the study population.

Characteristics	TB (n = 100)	Non-TB (n = 43)	*P value*
Age, median(range), yrs	22(20-30)	21(20-26)	0.02
Male Gender	98(98)	43(100)	0.87
Smoking History			
Non-smoker	50(50.0)	22(51.2)	0.46
Ex-smoker	3(3.0)	4(9.3)	
Current smoker	47(47.0)	17(39.5)	
BCG scar present	70(70.0)	32(74.4)	0.59
Sputum AFB smear			
Negative	99(99.0)	39(90.7)	<0.05
Non-productive	1(1.0)*	4(9.3)^†^	
Symptom present			
Cough	52(52.0)	29(67.4)	0.09
Sputum	39(39.0)	28(65.1)	<0.01
Hemoptysis	10(10.0)	8(18.6)	0.15
Peak BT(°C), mean ± SD	37.1 ± 0.8	37.6 ± 1.1	0.01
WBC(×10^3^cells/uL), mean ± SD	6.89 ± 1.85	8.33 ± 5.21	0.09
CRP(mg/dL), mean ± SD	2.02 ± 3.32	6.35 ± 8.98	<0.01
Final Diagnosis			
Culture-positive pulmonary TB	41(41.0)		
Clinical pulmonary TB	35(35.0)		
Extrapulmonary TB	24(24.0)		
Pleural TB	19(19.0)		
Lymph node TB	2(2.0)		
Other site	3(3.0)		
Pneumonia		30(69.8)	
Paragonimiasis		3(7.0)	
Empyema		2(4.7)	
Other diagnoses		8(18.6)	

Data are no.(%) of patients, unless otherwise indicated.

BCG, Bacille Calmette-Guerin; BT, body temperature; WBC, white blood cell; CRP, C-reactive protein; AFB, acid-fast bacilli; TB, tuberculosis

* *Mycobacterium tuberculosis *was isolated on bronchial washing fluid.

^† ^Four subjects include two with reactive lymphadenopathy, one with malignant lymphoma and one with paragonimiasis.

Five patients were not able to expectorate sputum. One of the five patients had MTB isolated in his bronchial washing fluid. Three of the five presented with cervical lymphadenopathy (LAP); one was diagnosed with malignant lymphoma on pathologic exam, and the other two were diagnosed with reactive LAP because the enlarged lymph nodes became smaller with resolution of symptoms during follow-up. The last of the five patients was diagnosed with paragonimiasis; this patient had a peripheral eosinophilia, eosinophil-dominant pleural effusion, and elevated serum-specific IgG antibody concentration for *P. westermani*. MTB was not isolated in the AFB culture of his pleural fluid.

### Diagnostic accuracy of TST, and QFT-IT

Of 142 patients with available TST results, the results were positive in 98 (69.0%), 93 of whom were diagnosed with active TB; the results were negative in 44 (31.0%) patients, six of whom was diagnosed with active TB. Of the 143 patients who underwent QFT-IT tests, five patients (3.5%) had an indeterminate result. The results of QFT-IT were positive in 95 patients (66.4%), 93 of whom were diagnosed with active TB; the results were negative in 43 (30.1%), seven of whom were diagnosed with active TB (Table 2).

**Table 2:** The results of TST and QFT-IT among active TB and non-TB disease.

	TST (n = 142*)		Total	QFT-IT (n = 138^†^)		Total
				
	positive	negative		positive	negative	
TB	93	6	99	93	7	100
Non-TB	5	38	43	2	36	38
Total	98	44	142	95	43	138

TB, tuberculosis; TST, tuberculin skin test; QFT-IT, QuantiFERON^® ^TB Gold In-Tube assay

*TST result was not available in one TB patient.

^† ^QFT-IT results were indeterminate in five non-TB patients.

The TST sensitivity for the diagnosis of active TB was 94% (95% CI, 87-98%), and the specificity was 88% (95% CI, 74-96%). The QFT-IT sensitivity was 93% (95% CI, 86-97%), and the specificity was 95% (95% CI, 81-99%). None of the four statistics was significantly different between the TST and QFT-IT, revealing excellent agreement between the two assays (κ = 0.83, with exclusion of five indeterminate results in QFT-IT, *P *< 0.001, Table 3).

**Table 3:** Diagnostic accuracy of TST and QFT-IT in the diagnosis of active TB

	TST (n = 142)	QFT-IT (n = 138)	*P *value
Sensitivity, % (95% CI)	94 (87-98)	93 (86-97)	0.79
Specificity, % (95% CI)	88 (74-96)	95 (81-99)	0.53
PPV, % (95% CI)	95 (88-98)	98 (92-99)	0.47
NPV, % (95% CI)	86 (72-94)	84 (69-93)	0.73
LR+ (95% CI)	8.1 (3.5-18.4)	17.7(4.6-68.2)	
LR- (95% CI)	0.07 (0.03-0.15)	0.07 (0.04-0.15)	
AUC (95% CI)	0.91 (0.85-0.97)	0.94 (0.89-0.99)	

PPV, positive predictive value; NPV, negative predictive value, LR+, positive likelihood ratio; LR-, negative likelihood ratio; AUC, area under curve; TST, tuberculin skin test; QFT-IT, QuantiFERON^® ^TB Gold In-Tube assay

### Clinical findings of the participants with indeterminate QFT-IT results

Five patients showed indeterminate QFT-IT results; all were non-TB cases. Four patients had bacterial pneumonia, and one patient had empyema. One patient showed severe lymphocytopenia (Patient 2), and three patients (Patients 1-3) showed low serum albumin concentrations (Table 4).

**Table 4:** Clinical findings of the participants with indeterminate QFT-IT results.

Patient No.	QFT-IT result	Age	BCG scar	WBC (cells/uL)	Lymphocyte count (cells/uL)	Serum total Protein (mg/dL)	Serum albumin (mg/dL)	TST (mm)	AFB Smear/Culture in sputum examination	Radiologic finding	Diagnosis
1	Indeterminate	22	Yes	10,390	1,039	6.4	3.3	0	-/-	Multifocal consolidations	Pneumonia
2	Indeterminate	21	Yes	28,180	366	5.9	3.2	0	-/-	LLL consolidation	Pneumonia
3	Indeterminate	20	Yes	7,110	683	5.3	2.8	0	-/-	Multifocal consolidations	Pneumonia
4	Indeterminate	23	Yes	10,620	1,296	7.6	4.2	0	-/-	RML, RLL consolidations	Empyema
5	Indeterminate	20	Yes	9,460	1,296	8.1	4.2	0	-/-	RLL consolidation	Pneumonia

QFT-IT, QuantiFERON^® ^TB Gold In-Tube assay; BCG, Bacille Calmette-Guerin; WBC, white blood cells; TST, tuberculin skin test; AFB, acid-fast bacilli; RML, right middle lobe; LUL, left upper lobe; LLL, left lower lobe; RLL, right lower lobe.

## Discussion

Our study showed that both the TST and QFT-IT had high sensitivity and specificity in the diagnosis of active TB among young military personnel in South Korea. The specificity for both tests was higher than that previously reported in South Korea [7,21], and that of the TST (88%) was slightly lower than that of the QFT-IT (95%); however, the difference was not statistically significant (*p *= 0.53).

There are two possible explanations for the higher sensitivities and specificities compared with those in previous reports. First, false-positive TST reactions due to BCG vaccination in infancy become minimal in young adults. In a review of 24 published studies regarding the effect of BCG vaccination on TST, only 1% of subjects vaccinated as infants were TST-positive if tested ≥10 years after BCG [15]. The prevalence of a positive TST has also been correlated with the number of BCG scars, a proxy for older age when last vaccinated [22,23]. BCG revaccination in individuals 12 to 13 years of age was discontinued in South Korea in 1997 based on a WHO recommendation [24]. Of the 143 patients enrolled in this study, only two patients had two BCG scars. Second, this study enrolled young and immune-competent adults. The median age of previous studies that reported lower specificities was 55 years [7]. The sensitivities of the IGRA are known to decrease significantly with older age [8], HIV infection [25], and chronic renal failure [26].

Previous studies in which the IGRA had a higher sensitivity than that of the TST [14,27] suggest that the IGRA may be more useful in the diagnostic exclusion of active TB. In a recent meta-analysis of IGRA for detecting active TB, the pooled sensitivity of the QFT-IT (81%; 95% CI, 78-83%) among definitively confirmed TB cases was higher than that of the TST (70%; 95% CI, 67-72%) [13]. However, few studies have compared the performances of the TST and IGRA in young adults. In a Japanese study on the clinical utility of the QFT-2G test for elderly patients with active TB, the rates of positive TST results were significantly higher in younger patients (27% vs. 70%) [8]. We assumed that the sensitivity of the TST would be comparable to that of the QFT-IT in young, previously healthy adults, and the sensitivity of the TST (94%) and QFT-IT (93%) showed no statistically significant difference (*p *= 0.79) in this study. However, considering the difference in positive likelihood ratio between TST and QFT-IT(8.1 vs 17.7), TST is expected to be less useful than QFT-IT for ruling in active TB as the post-test probability falls below 50%.

Indeterminate QFT-2G results were more common in patients receiving immunosuppressive therapy, especially those with lymphocytopenia [28]. One patient had severe lymphocytopenia. Malnutrition may lower the sensitivity of the IGRA [14]. Three patients showed low serum albumin concentrations. The serum albumin concentration is a marker of nutritional status; these patients may have been suffering from malnutrition, which could explain these indeterminate results.

We used 10 mm as the TST cutoff considering the communal setting, relatively higher incidence in South Korea than in other developed countries, and large number of subjects with BCG scars (71.3%). In the case of residents in high-risk congregate settings and recent immigrants from high-prevalence countries, ≥10 mm of induration is recommended as the criterion for a positive TST [16,29]. Most tuberculin skin reactions affected by BCG are less than 10 mm [30]. Although this can affect the diagnostic accuracy of the TST, the sensitivity and specificity were 97% (95% CI, 91-99%) and 88% (95% CI, 74-96%), respectively, when we applied 5 mm as the TST cutoff. Therefore, the change would be minimal if a cutoff of 5 mm were used in this study.

There are a few limitations in this study. First, the study population and clinical setting in this study were quite selective, and it is difficult to generalize this result to other situations. However, the study demonstrates the possibility that the TST and QFT-IT can play roles in determining TB disease for selected population. Second, comparisons with other age groups were not performed, and the diagnostic accuracy was evaluated for only one group. Third, the influence of BCG vaccination could not be evaluated because we did not know the accurate age of BCG vaccination of the subjects. However, school-age revaccination was abandoned in Korea 15 years ago when these subjects were 5 to 15 years old. For the past 15 years, BCG vaccination has been performed only at birth. Therefore, we can estimate that most subjects receive BCG vaccination at birth and that the effect on the TST is minimal.

## Conclusions

Both the TST and QFT-IT showed high diagnostic accuracy for discriminating active TB from other pulmonary diseases. The diagnostic accuracy of these two tests did not differ significantly when applied to this clinical population of young, immunocompetent adults in whom neonatal BCG vaccination was common, there was no history of previous TB and in whom suspicion of TB was high. Therefore, in some selected populations, the TST and IGRA may be utilized as reliable aids to diagnose active TB, although the diagnosis should be confirmed by microbiologic, radiologic, and clinical methods.

## List of abbreviations

TB: tuberculosis; HIV: human immunodeficiency virus; AFB: acid-fast bacilli; TST: tuberculin skin test; QFT-IT: QuantiFERON^® ^TB Gold In-Tube assay; QFT-2G: QuantiFERON-TB-2 Gold; IGRA: interferon-γ release assay; LTBI: latent TB infection.

## Competing interests

The authors declare that they have no competing interests.

## Authors' contributions

JEL collected the data for this study, analyzed the data, and wrote the manuscript. HJK assisted with planning of this study. SWL planned this study, collected and analyzed the data, and wrote the manuscript. All authors read and approved the final manuscript.

## Pre-publication history

The pre-publication history for this paper can be accessed here:

http://www.biomedcentral.com/1471-2334/11/96/prepub
